# Multiple introductions and gene flow in subtropical South American populations of the fireweed, *Senecio madagascariensis*(Asteraceae)

**DOI:** 10.1590/1678-4685-GMB-2015-0167

**Published:** 2016

**Authors:** Geraldo Mäder, Luana Castro, Sandro Luis Bonatto, Loreta Brandão de Freitas

**Affiliations:** 1 Laboratório de Evolução Molecular, Departamento de Genética, Universidade Federal do Rio Grande do Sul, Porto Alegre, RS, Brazil; 2 Laboratório de Genômica e Biologia Molecular, Pontifícia Universidade Católica do Rio Grande do Sul, Porto Alegre, RS, Brazil

**Keywords:** Microsatellites, ITS, Pampas, weed, spread

## Abstract

Non-indigenous plants exhibit different attributes that make them aggressive competitors with indigenous plants and serious threats to biodiversity.*Senecio madagascariensis* (fireweed, Asteraceae), a native from southern Africa, is a strong competitor in agricultural activities and has toxic alkaloids that may result in high cattle mortality. In Brazil, this weed was collected for the first time in 1995 and has since spread quickly throughout the Pampas region. To better understand the invasion of the fireweed in South America, we used a genetic characterization with internal transcribed spacer (ITS) and microsatellite markers. Based on the ITS data, the southern Brazil populations of *S. madagascariensis* shared genetic homology with samples taken from the Hawaiian Islands and South Africa. Microsatellite analysis showed the genetic diversity split in two clusters, perhaps intimating the independent introduction of each species into South America. Although fireweed was introduced recently in southern Brazil, the considerable levels of genetic diversity, gene flow, and inbreeding may indicate success in the species establishment in this environment.

## Introduction

Invasive plants have different attributes and mechanisms that allow their dispersal and establishment in new locations (López-García and Maillet, 2005; Murray and Phillips, 2010). They may be highly aggressive competitors for light and soil nutrients with indigenous plants (Le Roux *et al*., 2006). Furthermore, the absence of herbivores and pathogens may lead to the unbridled growth of invasive plants (Keane and Crawley, 2002; Allendorf and Lundquist, 2003). These plants produce a direct impact on the natural ecosystems and are serious threats to global biodiversity (Sala *et al*., 2000; Allendorf and Lundquist, 2003; Strauss *et al*., 2006). Studying the early stages of an invasion may reveal additional data on the processes of microevolution (Mooney and Cleland, 2001;Sakai *et al*., 2001;Lee, 2002; Barrett *et al*., 2008; Rosenthal *et al*., 2008).

Blackburn *et al*. (2011)proposed four main stages in successful invasions: first, a species disperses to a new geographic region (transport and introduction); third, it establishes populations in the new environment (establishment); and finally, it reproduces successfully (spread). To progress through each of these phases, invasive species must overcome barriers that are directly linked to genetic processes, such as the founder effect, genetic drift, gene flow and natural selection. Small founder populations have a limited genetic pool due to the founder effect and genetic drift, which may hinder their establishment in new environments (Sakai *et al*., 2001). Historically, phenotypic plasticity has been seen as a potentially important mechanism for the success of colonization in environmentally diverse areas and may play a role in invasion (Chun *et al*., 2009). Adaptive plasticity may contribute to invasive ability by allowing the acclimation of invasive pre-adapted genotypes to diverse environments, as well as buffering against existing genetic variation from selection, thereby reducing the necessity for local adaptation (Sultan, 2003; Thébault *et al*., 2011). Therefore, adaptive plasticity may lead to phenotypic homeostasis (tolerance) in fitness, which can be important to successful invasion (Rejmánek, 2000; Alpert and Simms, 2002). Another factor that may increase the likelihood of success for an invasive species is the decrease in the founder effect through multiple sources of introduction, as observed in several studies (Novak, 2007; Chun *et al*., 2009; Ghabooli*et al*., 2011). High genetic diversity can be beneficial on both ecological and evolutionary timescales: in the short term, high diversity has been shown to improve colonization success (Crawford and Whitney, 2010; Bock*et al*., 2015). Newly introduced populations often experience a genetic bottleneck, which can have potentially dramatic consequences for their evolution and survival in the new site. However, multiple introductions often restore the lost diversity, frequently resulting in higher levels of variability than in the native range because of admixture of genetically different sources of variation (Bock *et al*., 2015)

Trade and travel are primarily responsible for the introduction of alien organisms across the globe (Keller *et al*., 2011). Unfortunately, terrestrial species are inadvertently transported in shipping containers (Lounibos, 2002), facilitating the rapid spread of invasive organisms (Kaluza *et al*., 2010). Often, weeds (frequently associated with seeds) are recovered as unknown contaminants in baggage. Although biological invasions are serious threats to biodiversity, they are opportunities to understand fundamental ecological and evolutionary processes (Sax *et al*., 2007).

*Senecio madagascariensis* Poir., widely known as fireweed, is a member of the Asteraceae family, native to southern Africa (Le Roux *et al*., 2006). It is characterized as an annual herb, sometimes behaving as a perennial, under favorable conditions. Plants of this species when isolated appear to be fully self-compatible (Le Roux *et al*., 2010). They produce large numbers of seeds that disperse long distances by wind and are strong competitors for agricultural resources, while the toxic alkaloids they contain may result in death when ingested by animals (Le Roux*et al*., 2006). Fireweed was accidentally introduced to Australia c.1918 (Dormontt *et al*., 2014) and to the Hawaiian Islands in the early 1980s (Le Roux *et al*., 2006). In these locations, this weed competed strongly with existing pasture flora, ultimately leading to the deterioration of pastures (Le Roux*et al*., 2006). In Australia, Sindel *et al*. (1998) reported that the species has a high degree of competitiveness as a weed, causing great economic losses due to the mortality of cattle.

In Argentina, the first specimen of *S. madagascariensis* was collected in 1940; the current populations are entirely diploid (López *et al*., 2008). In Brazil, fireweed was first identified in 1995 (Matzenbacher and Schneider, 2008), and in recent years, it has spread quickly throughout the Pampas in southern areas (see Figure S1). In other parts of Brazil, the weed does not appear to grow below the southern latitude of 29°S. More recently, there is evidence that fireweed, together with other*Senecio* species, has caused great economic losses to livestock in southern Brazil, with 45,500 animals lost annually (Karam *et al*., 2011). Cattle production has existed in the Pampas region since the 17^th^ century and is the most important environmental change to have occurred in recent decades due to agricultural expansion in South America (Overbeck*et al*., 2007). The effective management of invasive plant species may be facilitated by further research into the biological processes that were challenging to clarify or quantify under field conditions prior to recent advances in molecular genetics. To contribute to a better understanding of the invasion of *S. madagascariensis* in southern Brazil, we aimed a) to clarify the invasion history including the origin of introduced genotypes and the possibility of multiple introduction events; b) to determine the genetic diversity and the structure of the invasive populations; and c) to examine the gene flow among populations.

## Materials and Methods

### Sample collection and DNA extraction

A total of 307 *S. madagascariensis* individuals were sampled from 15 sites (hereafter referred to as populations) covering the entire geographical range of the distribution for this species in Brazil and Uruguay (Table 1; Figure 1). We collected 20–30 individuals per site, where practical, and the minimum distance between two collection points was 25 km. All populations were found at elevations below 200 m in the Pampas region, preferentially in lowlands close to sea level. The TOR population (~29°S) was the northernmost *S. madagascariensis* in Brazil. Vouchers were deposited in the ICN Herbarium (at the Universidade Federal de Rio Grande do Sul, Porto Alegre, RS, Brazil), and young leaves were carefully collected in the field for genetic analysis. After drying on silica gel, the leaves were frozen in liquid nitrogen and ground into a fine powder. Subsequently, DNA was extracted from the powdered leaves as described by Roy *et al*. (1992).

**Table 1:** Genetic diversity and collection sites information for the Senecio madagascariensis populations sampled

Local information						ITS	Microsatellite diversity					
Population	*n* ^1^	*n* ^2^	Collection site	Geographical coordinates	Voucher*	Sequence types#	A	*A_R_*	Ho	He	*F_is_*	*P_A_*
URU	04	04	URU, Ciudad Del Plata	34°45' 53″S/56°24' 25'W	166599	S3;S4	3.00	2.71	0.412	0.577	0.316	2
POA	10	21	BRA, Porto Alegre	30°04' 01″S/51°07' 14″W	166600	S1–2; S6; S11–12	7.50	3.67	0.454	0.763	0.411	2
NSR	11	22	BRA, Nova Santa Rita	29°50' 45″S/51°16' 38″W	166601	S1–2; S6; S13; S17	8.13	3.48	0.377	0.731	0.490	7
RIG	18	24	BRA, Rio Grande, Povo Novo	31°56' 31″S/52°18' 39″W	166604	S1–2	5.88	2.94	0.352	0.652	0.465	3
VIA	13	21	BRA, Viamão	30°08' 15″S/50°52' 05″W	166605	S1–2	6.75	3.28	0.452	0.698	0.358	3
CPS	13	22	BRA, Capivari do Sul	30°08' 51'S/50°30' 31″W	166606	S1–2; S18	8.13	3.62	0.467	0.738	0.374	4
GLO	10	14	BRA, Glorinha	29°53' 25″S/50°42' 08″W	166607	S1–2	6.38	3.59	0.402	0.759	0.479	0
OSO	06	17	BRA, Osório	29°45' 05″S/50°12' 47″W	166608	S1–2; S19	6.13	3.31	0.477	0.710	0.337	1
TOR	08	26	BRA, Torres	29°18' 17″S/49°46' 21″W	169679	S1–2; S10	8.00	3.58	0.548	0.756	0.279	2
PLS	08	12	BRA, Palmares do Sul	30°25' 37″S/50°30' 04″W	169680	S1–2; S20	4.00	2.73	0.584	0.626	0.071	1
BAG	07	33	BRA, Bagé	31°17' 57″S/54°04' 46″W	170944	S1;S14	8.25	3.45	0.534	0.711	0.252	5
ELS	10	22	BRA, Eldorado do Sul	30°03'58″S/51°33' 27″W	166602	S1–2; S6; S7	7.50	3.34	0.597	0.722	0.177	6
BAR	11	21	BRA, Barra do Ribeiro	30°25' 22″S/51°28' 03″W	166603	S1–2; S9	8.13	3.67	0.451	0.764	0.417	2
MIL	04	18	BRA, Minas do Leão	30°08' 59″S/52°04' 18″W	170943	S1–2; S8	8.50	3.85	0.551	0.771	0.292	7
CHS	06	30	BRA, Cachoeira do Sul	30°16' 30″S/52°56' 13″W	166946	S1-2; S16	9.13	3.61	0.529	0.747	0.295	10
Total	139	307				Average	7.03	3.39	0.479	0.715	0.334	

Sample size (*n* ^1^ = ITS data;*n* ^2^ = SSR data); number of alleles per population (*A*); allelic richness (*A_R_*); average observed heterozygosity across loci (*He*); expected heterozygosity (*Ho*) and fixation index (*F_IS_*); private alleles (*P_A_*).

* ICN Herbarium, Department of Botany, Biosciences Institute, Universidade Federal do Rio Grande do Sul, Porto Alegre, Brazil.

# Sequence types of samples outside of South América: South África S1; Hawaii S2; Swaziland S21; Madagascar S22-26.

**Figure 1:**
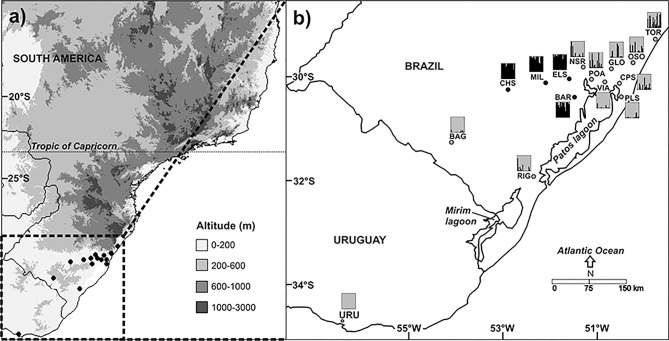
Collection sites. a) Map of South America highlighting the Brazilian state of Rio Grande do Sul and Uruguay. b) Colored dots indicate all collected populations of *Senecio madagascariensis* Poir. included in this study. The different shapes correspond to the clusters by STRUCTURE based on SSR data (rectangles; bars represent the membership coefficients (Q); K = 2) and NJ dendrogram/network (circles).

### Molecular population genetic analysis of ITS

For these analyses, we used 139 samples belonging to 15 populations (Table 1) and 24 GenBank sequences from the following regions: Australia (1); Argentina (1); Madagascar (8); South Africa (7); Swaziland (2); and the Hawaiian Islands (5), respectively (GenBank accession numbers JN789815, U93198, DQ322598-DQ322619). The internal transcribed spacers (ITS 1 and 2) were amplified using primers and reaction conditions described by Desfeux and Lejeune (1996). We used 10% dimethyl sulfoxide (DMSO) to exclude the presence of unstable templates (Buckler *et al*., 1997; Fuertes-Aguilar and Nieto-Feliner, 2003). All PCR products were purified using a 20% polyethyleneglycol (PEG) precipitation method (Dunn and Blattner, 1987). Sequencing was performed in a MegaBACE 1000 automatic machine using the ET Terminator Kit (GE Healthcare) following the manufacturer's protocol. The sequences were aligned manually using the GeneDoc program (Nicholas *et al*., 1997) and deposited in GenBank (accession numbers: JQ653968-JQ654097). Following Mäder *et al*. (2010), the heterozygous ITS sites were not included in the analysis.

We used DnaSP 5 (Librado and Rozas, 2009) to determine the sequence types. Basic sequence statistics, such as haplotype (*h*) and nucleotide diversity (π) (Nei, 1987), and the analysis of molecular variance (AMOVA;Excoffier *et al*., 1992) among collection sites using *F_ST_*(pairwise differences) were obtained in Arlequin 3.5.1.2 (Excoffier and Lischer, 2010). We estimated the evolutionary relationships among the sequences by the Median-Joining method (ε = 0; Bandelt *et al*., 1999) as implemented in Network 4.6 software. Sequences of S. inaequidens from GenBank were used as the outgroup (DQ322620 and DQ322621).

### Molecular population genetic analysis of microsatellite markers

We used all 307 sampled individuals (Table 2) to assess the genetic variability at eight nuclear microsatellite loci (SSR). Details of PCR protocols, isolation, characterization, and internal repeat structures of the *S. madagascariensis* microsatellite loci used in this study can be found in the study by Le Roux and Wieczorek (2007). In the present work, we analyzed only the eight loci also used by Le Roux *et al*. (2010). PCR amplifications were performed in 15-μl reaction volumes containing 2.5–5 ng of genomic DNA. Microsatellite genotyping was performed using a MegaBACE 1000 automated sequencer and the ET550-R Size Standards (GE Healthcare). Allele sizes were scored using *Genetic Profiler 2.2 software* (Amersham Biosciences). All microsatellite loci were screened for null alleles and large allele dropouts using MICRO-CHECKER 2.2.3 (Van Oosterhout *et al*., 2004).

**Table 2:** Diversity and neutrality indices in Senecio madagascariensis based on ITS data

Parameter	South America	Complete data set*
Sample size	139	161
No. sequence types	20	26
*π* (SD)	0.001 (0.001)	0.002 (0.002)
Genetic diversity (SD)	0.624 0.02	0.763 (0.017)
*F_ST_*	0.146 (*P* <0.001)	0.312 (*P* <0.001)

* South America samples plus sequences from South Africa (7); Swaziland (2);

Madagascar (8) and Hawaiian Islands (5).

*π* = Nucleotide diversity.

SD= Standard deviation.

Genetic diversity was estimated for each population in terms of the number of alleles (*A*) and allelic richness (*A_R_*); expected (*H_E_*) and observed (*H_o_*) heterozygosity, deviations of genotype frequencies from those expected under Hardy-Weinberg equilibrium (HWE); and the inbreeding coefficient (*F_IS_*) using Arlequin and FSTAT 2.9.3 (Goudet, 2001). Additionally, we obtained AMOVA data from the populations using*F_ST_* (pairwise differences) in Arlequin.

The ISOLDE program (part of the Genepop 4.1 package) was used to test the relationships between geographic and genetic (*F_ST_*) distances among populations, with the statistical significance assessed using a Mantel test with 10,000 permutations. To test the structure and to assess the scale of geographic differentiation among populations, we used STRUCTURE 2.3.3 (Falush *et al*., 2007) with a burn-in of 250,000, a run length of 1,000,000 *Markov Chain Monte Carlo* (MCMC), and a model allowing for admixture and correlated allele frequencies. We did not use *a priori*information on population origin. Five independent runs were performed by setting the number of populations (K) from 1 to 15, and an average likelihood value, L(K), was calculated for each K across all runs. Additionally, we calculated ΔK (Evanno *et al*., 2005) by taking into account the shape of the log-likelihood curve with increasing K and variance among estimates among multiple runs. The runs of individual ancestry coefficients were calculated by the average pairwise similarity of individual assignments across runs using CLUMPP 1.1.2 (Jakobsson and Rosenberg, 2007) and plotted using DISTRUCT 1.1 (Rosenberg, 2004). An unrooted Neighbor-Joining (NJ) dendrogram was constructed from the genetic distance (proportion of shared alleles) calculated with the Microsat program using MEGA 5.0 (Tamura *et al*., 2011). To verify the existence of migrants between populations, we ran 10,000 MCMC simulations per population in GeneClass2 (Piry *et al*., 2004) using the Lh/Lmax likelihood computation, where Lh is the likelihood of an individual being assigned to the population from which it was sampled and Lmax is the maximum likelihood for all populations considered. An individual was considered a migrant if the Lh/Lmax *P* value was below 0.01. Statistical comparisons among populations were performed using the paired Student's t-test, with significance being designated for P <0.05.

## Results

### Distribution

During the fieldwork, it was possible to observe the rapid spread of *S. madagascariensis*, particularly in the Rio Grande do Sul Brazilian state. Population growth was observed in different locations, where this fireweed was not registered previously by Matzenbacher and Schneider (2008) (OSO and TOR). In other localities (BAG and CHS), populations were noticeably more prevalent than the local vegetation, forming dense populations in the Pampas region (unpublished data based on fieldwork performed in the last six years, 2009–2014) during the flowering period of fireweed (see Figure S1).

### ITS data

**Figure 2:**
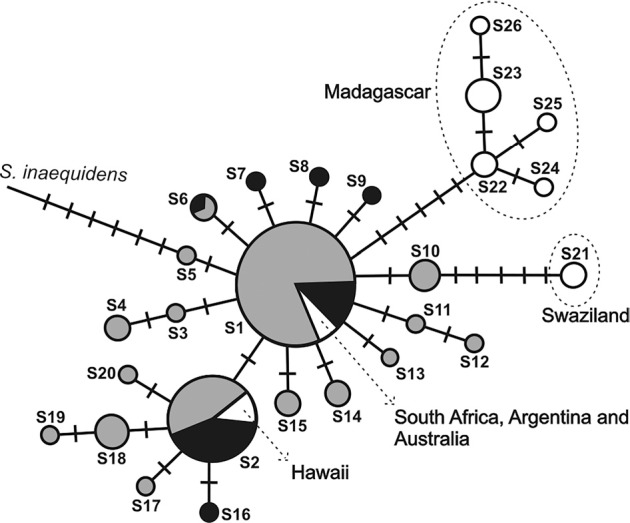
The evolutionary relationships of *Senecio madagascariensis* Poir. based on ITS sequence types obtained by median-joining network approach. Gray scale shading identify the geographic origin. Circle sizes are proportional to haplotype frequency. Crossed lines represent substitutions inferred in the branches.

The internal transcribed spacer ITS length varied between 665 and 666 base pairs (bp) in all analyzed samples, with 31 polymorphic sites in the alignment and one insertion/deletion event and 30 substitutions (11 transitions and 19 transversions). Among the 26 sequence types inferred by DNAsp, nucleotide diversity (π) was 0.0023 ± 0.002, while genetic diversity was 0.763 ± 0.017 (Table 2). Of the 26 types of sequences, 20 were sampled from the South American populations; therefore, the value of genetic diversity was similar to the total sample (0.624 ± 0.02) (Table 2). A moderate degree of genetic structure was observed (*F_ST_* = 0.312;*P* <0.001) in the global dataset when the populations were clustered into five geographical regions: South America, South Africa, Madagascar, Swaziland, and the Hawaiian Islands. We analyzed the 15 populations from South America independently from the other global populations (Table 1). *F_ST_*decreased to 0.146 (*P* <0.001), showing a reduction in the genetic structure. Median-joining network analysis verified the absence of a geographic structure in South American populations (Table 1; Figure 2). Among the South American populations, URU was the only population that did not show the central sequence type (S1), while S2 was observed in all populations except URU and BAG. Only RIG, VIA and GLO did not present exclusive sequence types. All South American populations had at least two sequence types. A prominent network feature was the relationship of the South American populations with the sequences from the Hawaiian Islands and South Africa (S1 and S2). The sequences of plants from Madagascar remained in an isolated cluster (sequence types S22-S26), and the Swaziland samples presented only one unique sequence type (S21) (Figure 2).

### Microsatellite marker

The eight microsatellite loci tested were polymorphic across all 307 individuals from the 15 populations. Across all individuals, the number of alleles per locus ranged from 10 (Se-194) to 44 (Se-136); within populations, the number of alleles ranged from 2 to 17 (mean = 7.03) and allelic richness ranged from 2.71 to 3.75 (mean = 3.39) (Table 1). Expected heterozygosity (*H_E_*) ranged from 0.577 to 0.771 (mean = 0.715), observed heterozygosity (*H_O_*) from 0.352 to 0.597 (mean = 0.456) and *F_IS_* ranged from 0.071 to 0.490 (mean = 0.334) across all loci (Table 1). Except for the Uruguayan population (URU), all populations showed a deficit of heterozygotes under Hardy-Weinberg Equilibrium (α = 0.05), and few null alleles were detected at very low frequencies.

**Figure 3:**
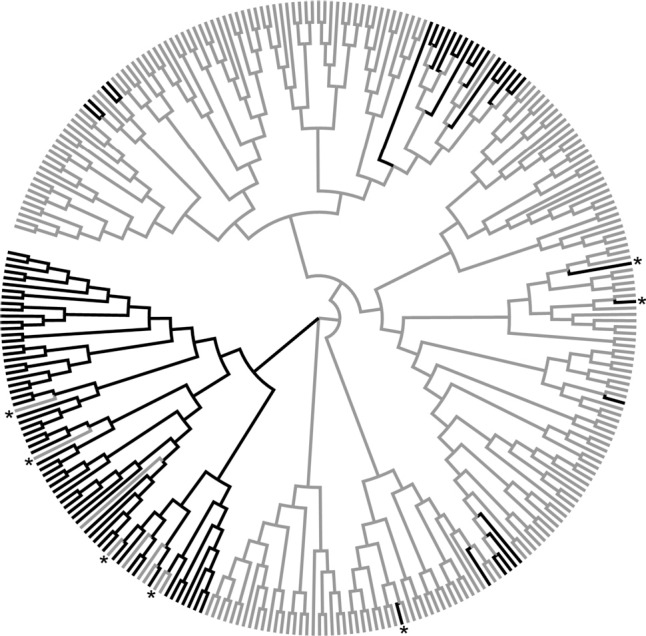
The unrooted Neighbor-Joining dendrogram based on the proportion of shared allele distances obtained by SSR genotypes. The branch colors indicate the two groups inferred by the STRUCTURE software. Black: Central group. Gray: Coastal group. *First-generation migrants among groups by estimates of dispersal in GeneClass2.

Diversity within population explained ca. 90% of the genetic diversity based on microsatellite (*F_ST_* = 0.103), similar to the results from the sequence data (ITS). Across fireweed populations, the Mantel test showed a moderately significant positive correlation between genetic and geographic distances (*r* ^2^ = 0.37; *P*<0.001) (see, Figure S2). The results of Bayesian cluster analyses revealed the presence of structured genetic diversity among groups. Following the method of Evanno *et al*. (2005), the model-based clustering method implemented in STRUCTURE found two distinct major genetic clusters (K) (Figure 1). A cluster including four populations (ELS, BAR, MIL and CHS) that presented more continental geographic distribution (latitude approximately 30° S) was named "Central Group". The second cluster was composed of the remaining populations, found in the Coastal Plain region (except for BAG), and was named "Coastal Group". We detected some admixture between the two clusters (*e.g*., POA, NSR, GLO, TOR; Figure 1). The unrooted NJ dendrogram (Figure 3) distinguished two clades of samples, corresponding to the same groups obtained by STRUCTURE, and similarly showed admixture between Central and Coastal groups. Some samples were found in the clade of the opposite group (Figure 3). More precisely, 30 individuals of the Central Group were found in the Coastal clade and 10 individuals of the Coastal Group positioned in the Central clade. Furthermore, AMOVA showed that 38.7% of the variation found refers to the difference between Central and Coastal Groups (*F_ST_* = 0.387). On average, populations from the Central Group differed significantly from Coastal Group populations in terms of heterozygosity (*Ho*), inbreeding (*F_IS_*) and levels of differentiation among populations (*F_ST_*). However, there were no significant differences in observed allelic richness (*A_R_*) or unbiased gene diversity (*Hs*) between the two groups (Student's paired*t*-test) (Table 3).

**Table 3:** Statistical comparison of allelic richness (*A_R_*), unbiased gene diversity (*H_s_*), observed heterozygosity (*H_o_*), inbreeding coefficient (*F_IS_*) and levels of differentiation among populations (*F_ST_*) for the two genetic groups of *Senecio madagascariensis*

	n	A_R_	Hs	Ho*	F_IS_ *	F_ST_ *
Coastal group	216	3.305	0.739	0.460	0.348	0.073
Central group	91	3.616	0.794	0.532	0.295	0.103

* *P* <0.05.

n = sample size.

Contemporary estimates of dispersal calculated in GeneClass2 identified 38 of 307 individuals as potential 'first-generation migrants' (*P* = 0.01). However, given the low genetic structure observed between pairwise populations, the results based on the identification of immigrants should be interpreted with caution because a decrease in genetic differentiation and limited number of loci reduces the ability to correctly identify immigrants. Therefore, we focused on overall patterns of potential migration. Not surprisingly, many of the migrants between Central and Coastal groups correspond with those individuals that show a distinct population pattern in the dendrogram (indicated by asterisks in Figure 3) and STRUCTURE analysis.

## Discussion

### Origin and preadaptation

Based on the ITS data, the South American populations of fireweed were more closely related to the samples from the Hawaiian Islands and South Africa (sharing the more frequent sequence types, S1, S2), while samples from Madagascar and Swaziland were unique and distant from the others (Figure 2). This suggests that the South American populations most likely originated in South Africa, or alternatively, originated in another country where fireweed is alien (*e.g*., Australia or the Hawaiian Islands), constituting a secondary invasion. Several works have attributed the arrival of invasive plants via cargo ships from South Africa (McCullough *et al*., 2006; Lachmuth *et al*., 2010; Keller *et al*., 2011). The first hypothesis is the most probable given the large number of trade routes between sub-tropical South America and southern Africa, while the same is not true of Australia or the Hawaiian Islands (Kaluza *et al*., 2010). Furthermore, *S. madagascariensis* was only just recently introduced into South America, so there are no dense populations in Australia or on the Hawaiian Islands (Le Roux*et al*., 2006), suggesting the difficulty in its migration to a new continent. The populations of fireweed in subtropical South America are located in areas with similar latitude (usually 29–32°S) and altitude (<200 m, predominantly at sea level, Figure 1). Their indigenous populations in South Africa include the harbor area of the KwaZulu-Natal region. This is the indicated source of the invasion into the Hawaiian Islands according to Le Roux *et al*. (2006). This putative origin may have facilitated the adaptation of these aliens in Brazil, which may be pre-adapted to environments with similar climatic conditions, a great advantage in terms of general survival according to Thébault *et al*. (2011).

### Genetic variability and structure

The ITS data showed many sequences in South American populations of fireweed. Over 75% of the sampled sequence types (20 of 26) were found in South America. However, the most thorough comparison is the quantification of the genetic variability of indigenous *S. madagascariensis* populations. Although the ITS sequences of *S. madagascariensis* have exhibited low variability when compared with other groups of plants (*e.g*., Nettel and Dodd, 2007; Yamaji *et al*., 2007; Mäder *et al*., 2010), the difficulty in finding informative molecular markers in*Senecio* has been previously observed (*e.g*., Comes and Abbott, 2001; Pelser *et al*., 2007), as well as in other plants outside their natural habitat (Young and Murray, 2000; Yu *et al*., 2014). Similarly, several studies, such as those conducted by Lavergne and Molofsky (2007) and by Ghabooli *et al*. (2011) found low*F_ST_* values in invasive populations. The potential high number of private sequence types observed in invasive populations is probably due to the small number of individuals from their region of origin included in this study. Nevertheless, some private sequence types might have originated *in loco*, after the invasion, since a majority of haplotypes differs from each other by a single mutational step. Unfortunately, a more accurate conclusion requires large-scale sequencing in the region to which fireweed is indigenous (southern Africa), and this information is not available. Therefore, due to insufficient variability, the ITS analysis did not allow us to explore the network relationships among South American populations. Instead, the relationships were analyzed on a global scale, allowing the differentiation of the indigenous populations in southern Africa (South Africa, Swaziland and Madagascar) (Figure 2).

Microsatellite analysis revealed levels of genetic diversity similar (number of alleles, allelic richness and heterozygosity observed) to those obtained from the Hawaiian populations (Le Roux *et al*., 2010). However, these values were lower than those observed in populations from Australia and South Africa (Dormontt *et al*., 2014). Nevertheless, as observed by Dormontt *et al*. (2014), the reduction in the genetic diversity of fireweed does not appear to have hindered its spread, perhaps due to high levels of phenotypic plasticity, changes in gene expression or the 'adequacy' of diversity for populations in adapting to the new conditions. The two clusters observed in the STRUCTURE analysis (Figure 1) and the NJ dendrogram (Figure 3), beyond the distinctive characteristics listed in Table 3, suggest at least two independent introduction events. These results suggest that invasive populations in South America have a genetic diversity that may be related to multiple introduction events, and this may be related to shipping traffic from southern Africa, as previously mentioned. Several studies have shown, using historical and molecular data, that many invasive weeds have significant genetic diversity as a result of multiple introductions; for example: *Alliaria petiolata* (Bieb.) Cavara and Grande analyzed by Durka *et al*. (2005) and *Phalaris arundinacea* L. in Lavergne and Molofsky (2007). Alternatively, we may not reject that the two observed groups are related to a process of genetic drift following the introduction of fireweed into the Pampas. The Mantel test indicates a positive correlation between genetic and geographic diversity; however, due to the recent colonization of fireweed in subtropical South America, it is unlikely that this is related to patterns of structuring by some type of barrier to gene flow, but instead is caused by the founder effect of each population.

### Spread and gene flow

Although fireweed was introduced recently into southern Brazil, the field observations in recent years suggest that this weed has already reached the current spreading stage. This rapid success may be related to high production, seed dispersal and preadaptation rather than to early aliens (Le Roux *et al*., 2010;Thébault *et al*., 2011). In addition, we found a proportionally higher number of migrants than reported by Le Roux *et al*. (2010) in the Hawaiian Islands. This difference can be explained by the greater ability of pollen and seeds to disperse in an environment with continuous lowlands, as in the Pampas region (Chaneton and Lavado, 1996). Another factor that may be contributing to the rapid dissemination of *S. madagascariensis* in the Pampas region is the considerable level of inbreeding observed here. This reproductive feature facilitates rapid population increase and spread. Additionally, Le Roux*et al*. (2010) showed that moderate levels of inbreeding are commonly observed in other alien species (*e.g*.,Young and Murray, 2000; Yu *et al*., 2014). Inbreeding may be an indirect consequence of inter-breeding between close relatives due to the founder effect and/or excessive self-fertilization (Le Roux *et al*., 2010). The moderate levels of inbreeding produced heterozygosity values lower than those expected and may provide some evidence of self-fertilization of *S. madagascariensis* in the Pampas region. In contrast, the gene flow among populations can influence a rapidly increasing population in preventing genetic drift and inbreeding depression (Le Roux*et al*., 2010).

### Impacts in the Pampas region

*Senecio madagascariensis* has been very effective in its dispersal and establishment in new locations in the Pampas region. The considerable levels of genetic diversity, gene flow and inbreeding indicate that this species has great potential for successful establishment in new environments in southern Brazil. Fireweed is quickly expanding geographically and in population density. Additionally, fireweed is an increasingly significant threat to indigenous species and livestock production in the Pampas region. Researchers aim to control this and other *Senecio* species in the Pampas region (Karam *et al*., 2011, 2013) due to the severe danger that these plants represent for cattle. Our results may underpin future studies about this weed in subtropical South America and also in other locations around the world.

Thus, for a better understanding of the invasion of *Senecio madagascariensis* into the Pampas region it is still important to investigate more molecular markers and other populations from Uruguay and Argentina. In this regard, the EST collection performed by Prentis *et al*. (2010) provides many initial candidate-gene markers that can help to answer a number of leading hypotheses in invasion biology.

## Supplementary Material

The following online material is available for this article:

Figure S1 - *Senecio madagascariensis* in Bagé.

Figure S2 - Relationship between geographical (km) and genetic (*F_ST_*) distances.

This material is available as part of the online article from http://www.scielo.br/gmb
