# Supplementary figures and images for: Multiple introductions and gene flow in subtropical South American populations of the fireweed, Senecio madagascariensis(Asteraceae)
Source: Genet Mol Biol. 2016 Jan-Mar;39(1):135–44. doi: 10.1590/1678-4685-GMB-2015-0167 (PMC4807391; doi:10.1590/1678-4685-GMB-2015-0167)

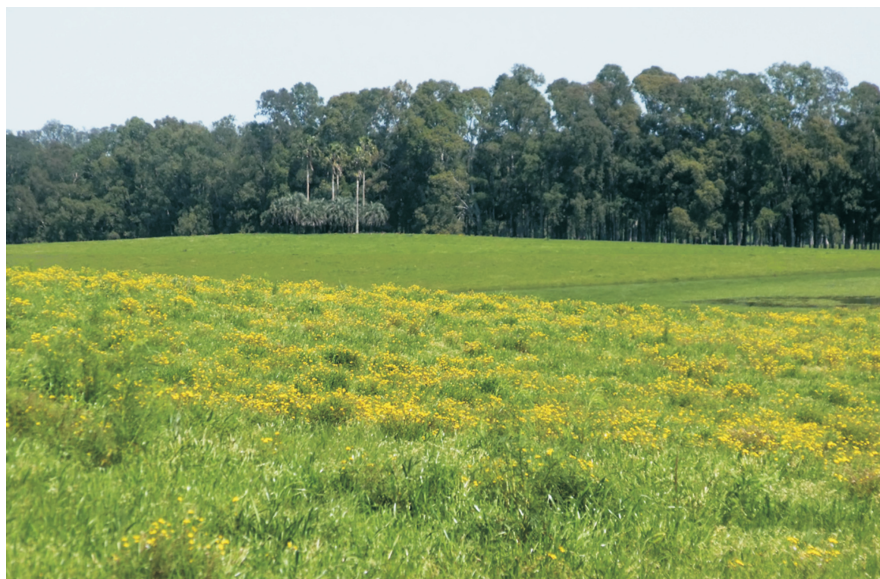

**Figure S1** - *Senecio madagascariensis* forms dense populations in Bagé, Brazil.

Supplement: Supplementary file 1 [file 1415-4757-gmb-39-1-135-Suppl01.pdf]
