# Supplementary material for: Multiple introductions and gene flow in subtropical South American populations of the fireweed, Senecio madagascariensis(Asteraceae)
Source: Genet Mol Biol. 2016 Jan-Mar;39(1):135–44. doi: 10.1590/1678-4685-GMB-2015-0167 (PMC4807391; doi:10.1590/1678-4685-GMB-2015-0167)
Supplement: Supplementary file 2 [file 1415-4757-gmb-39-1-135-Suppl02.pdf]

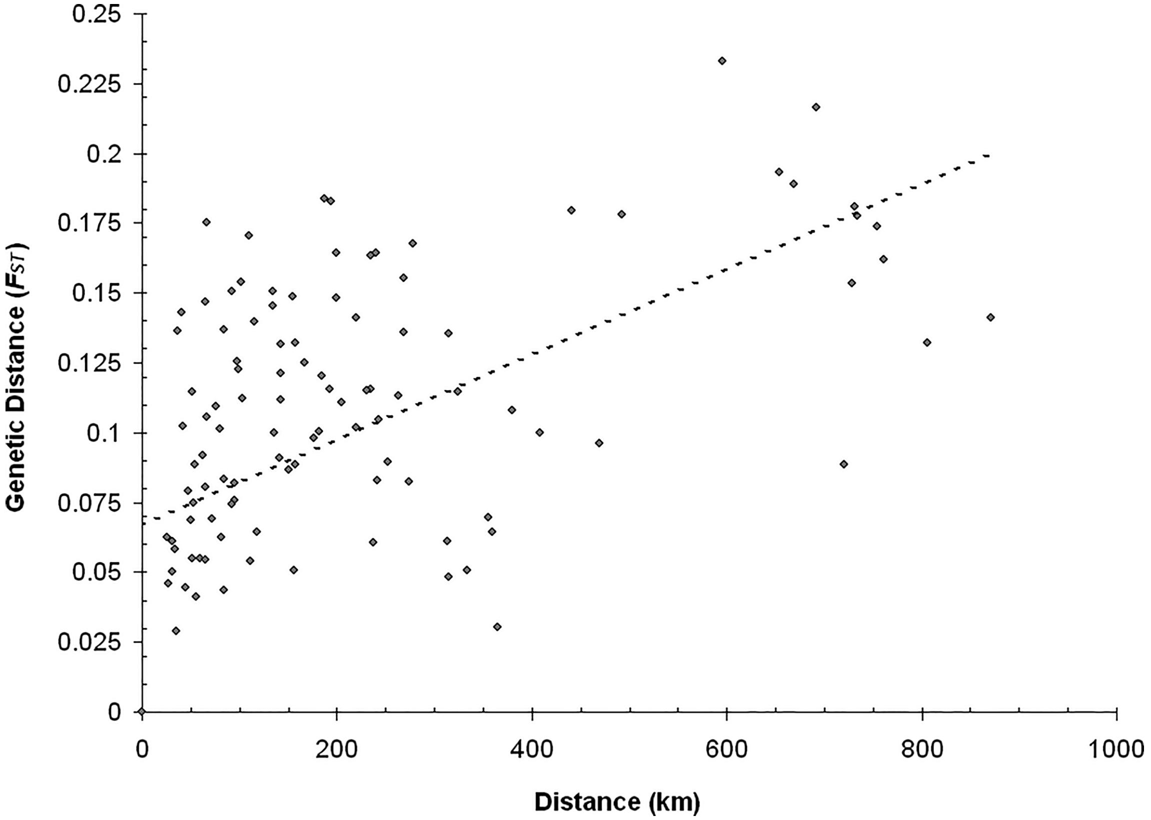

Figure S2 - The relationship between geographical (km) and genetic (F_{ST}) distances among all populations of *Senecio madagascariensis* Poir. included in this study by regression analysis ($r^2 = 0.57$; P <0.001).
